# Charcot Marie Tooth Disease. A Single Disorder?

**DOI:** 10.3390/ijms19123807

**Published:** 2018-11-29

**Authors:** Michel Fontés

**Affiliations:** C2VN, AIX Marseille Université, INRA 1260—INSERM 1263, 13007 Marseille, France; michel.fontes@univ-amu.fr; Tel.: 33-491-32-443

## 1. Introduction

Peripheral neuropathies are subdivided into acquired and hereditary transmitted disorders. Among hereditary peripheral neuropathies, the most frequent is Charcot-Marie-Tooth disease (CMT). We will describe below, in detail, this disorder and its different forms.

Charcot-Marie-Tooth disease constitutes a clinically and genetically heterogeneous group of hereditary motor and sensory peripheral neuropathies. On the basis of electrophysiologic properties and histopathology, CMT has been divided into primary peripheral demyelinating (type 1) and primary peripheral axonal (type 2) neuropathies. The demyelinating neuropathies classified as CMT type 1, also known as HMSN I, are characterized by severely reduced motor nerve conduction velocities (NCV) (less than 38 m/s) and segmental demyelination and remyelination with onion bulb formations on nerve biopsy. The axonal neuropathies classified as CMT type 2, also known as HMSN II, are characterized by normal or mildly reduced NCVs and chronic axonal degeneration and regeneration on nerve biopsy. Among the CMT1 group, there are X-linked, autosomal dominant and autosomal recessive forms of CMT.

The typical presenting symptom is a weakness of the feet and ankles. The initial physical findings are depressed or absent tendon reflexes with a weakness of foot dorsiflexion at the ankle. The typical affected adult has a bilateral foot drop, symmetrical atrophy of muscles below the knee (stork leg appearance), pes cavus, atrophy of intrinsic hand muscles, especially the thenar muscles of the thumb, and absent tendon reflexes in both upper and lower extremities. The life span is not decreased [1].

## 2. Prevalence of CMT (Charcot-Marie-Tooth) Subtypes

There are only a few epidemiologic studies on the prevalence of CMT disease. The most generally accepted is the study by Skre published in 1974 [2]. A major reason is probably the heterogeneity of this disorder. Actually, about 80 genes presenting mutations and a CMT phenotype have been recorded. Therefore, the CMT phenotype is not homogenous and could be defined in a general term: “hereditary peripheral neuropathies”, whatever the cause is. We will describe below, the estimated prevalence of the CMTX1, a specific form of CMT disease.

CMT is also known as hereditary motor and sensory neuropathy (HMSN). Hereditary motor neuropathy (HMN) and hereditary sensory neuropathy (HSN) are related disorders and can also be considered as part of the CMT family. The most reliable evaluation of CMT prevalence is one affected person in 2500 [2]. Over 80 causative genes of CMT have been identified and many more remain unknown. The natural history of these various forms of CMT remains poorly understood, at least in part, because these are rare disorders and individual centers do not follow enough patients to perform natural history studies. Furthermore, validated clinical instruments for measuring disease severity have become available only recently and have not yet been employed in many of the rare CMT subtypes. The Inherited Neuropathies Consortium (INC) is a member of the Rare Diseases Clinical Research Network (RDCRN) and was created in part to perform natural history studies in CMT. Quantifiable clinical data add to the literature in providing the clinical severity of a variety of CMT subtypes and also act as a baseline for a longitudinal natural history study of CMT subtypes, a prerequisite for clinical trials.

From a recent study of the consortium published in 2015 [3], the frequency of different CMT subtypes ranged from 62% of patients with a genetic diagnosis for the most frequent subtype (CMT1A) up to 0.1% for CMT1D.

## 3. Different Genes, Different Proteins, Different Functions

Mutations in more than 80 different genes cause CMT. Below are listed the genes of known functions that could be regrouped in different sections:

### 3.1. Genes Involved in Cell Division

PMP22, the gene involved in CMT1A, was first identified as Gas3 in a screen of genes involved in growth arrest [4]. Histological analysis demonstrated that CMT1A is frequently associated with an abnormal number of Schwann cells (SC) between two Ranvier nodes (normally only one). This indicates that anomalies in the PMP22 expression have an impact in the growth arrest of Schwann cells [5,6,7]. Exploration of the cellular and rodent animal models showed that anomalies of myelination occurred in early phases of SC differentiation when the myelination of axons starts [8,9]. This indicates that anomalies of growth arrest impair SC terminal differentiation [10]. This is strengthened by observations that anomalies in nerve conduction velocity in youths, before full myelination and clinical signs appeared.

Another example regards mutations in Gjb1 (a gene coding for Connexin 32 CX32) that are involved in CMTX1 phenotype. A screen for genes potentially involved in the stability of mitoses, as well as observations in a mouse model or in cells from CMTX1 patients, demonstrated that Cx32 is involved in mitotic stability [11].

### 3.2. tRNA Synthetases

Aminoacyl-tRNA synthetases (ARSs) are ubiquitously expressed enzymes responsible for charging tRNAs with their cognate amino acids, therefore they essential for the first step in protein synthesis. Mutations in the majority of the 37 nuclear-encoded human ARS genes have been linked to a variety of recessive and dominant tissue-specific disorders. Current data indicate that impaired enzyme function could explain the pathogenicity, however not all pathogenic ARSs mutations result in deficient catalytic function; thus, the consequences of mutations may arise from other molecular mechanisms.

The peripheral nerves are frequently affected, as illustrated by the high number of mutations in tRNA synthetases causing Charcot-Marie-Tooth disease (CMT) but it remains particularly unclear what the cause of the high degree of tissue specificity could be. Various noncanonical functions of ARSs have become increasingly interesting. Understanding why peripheral nerves are predominantly affected will open potential therapeutic targets for a larger group of CMT patients; however, further research is still needed. For a review see Reference [12].

### 3.3. Mitochondrial Genes

Mitochondrial dysfunction plays a relevant role in the pathogenesis of neurological and neuromuscular diseases. Mitochondria may be involved as a primary defect of either the mtDNA or nuclear genome encoded subunits of the respiratory chain. Inherited peripheral neuropathies are frequently associated with abnormal mitochondrial network dynamics. As an example, mutations in the MFN2 gene cause the most frequent form of the autosomal dominant axonal Charcot-Marie-Tooth disease, CMT2A [13]. MFN2 is a GTPase involved in mitochondrial fusion processes [14,15]. Moreover, GDAP1 has been recently related to the mitochondrial fission in mammalian cells [16] and, interestingly, mutations in the GDAP1 gene are the cause of the most common form of autosomal recessive CMT, either axonal or demyelinating [17,18]. This gene encodes a member of the ganglioside-induced differentiation-associated protein family, which may play a role in a signal transduction pathway during neuronal development. These and other disorders are the most recent instances of diseases related to mitochondrial abnormal motility, fusion, and fission. the pathomechanisms underlying these disorders probably include a complex relationship between mitochondrial dynamics and transport across the axon. However, although biochemical functions are different, mutations in these genes result in anomalies in mitochondrial dynamics.

### 3.4. Myelin Compaction

Mutations in Myelin Protein Zero (MPZ) cause Charcot-Marie-Tooth type 1B [18]. Myelin protein zero (P0) is a membrane glycoprotein encoded by the MPZ gene. P0 is a major structural component of the myelin sheath in the peripheral nervous system (PNS) that accounts for over 50% of all proteins in the peripheral nervous system, making it the most common protein expressed in the PNS.

Myelin protein zero consists of an extracellular N-terminal domain, a single transmembrane region, and a smaller positively charged intracellular region. Its cytoplasmic domain is highly positively charged but presumably does not fold into a globular structure. The extracellular domain is structurally similar to the immunoglobulin domain and therefore the protein is considered as belonging to the immunoglobulin superfamily.

It is postulated that myelin protein zero is a structural element in the formation and stabilization of peripheral nerve myelin [19,20]. Myelin protein zero is also hypothesized to serve as a cell adhesion molecule, holding multiple layers of myelin together. When a myelinating cell wraps its membrane around an axon multiple times, generating multiple layers of myelin, myelin protein zero helps keep these sheets compact. It does so by holding its characteristic coil structure together by the electrostatic interactions of its positively charged intracellular domain with acidic lipids in the cytoplasmic face of the opposite bilayer and by the interaction between hydrophobic globular ‘heads’ of adjacent extracellular domains. Most patients with the CMT1B present in two phenotypic groups: one with extremely slow nerve conduction velocities and the onset of symptoms during the period of motor development; and a second with normal or near normal nerve conduction velocities and the onset of symptoms in adults.

### 3.5. Transcription Factors

Mutations affecting two transcription factors, EGR2/Krox20 [21] and Sox10 [22], have been found in CMT. EGR2/Krox20 is a zinc finger-containing protein and null mutant mice for this protein do not develop peripheral nerve myelin. This is consistent with several recent studies suggesting a broad function of EGR2/Krox20 in the regulation of Schwann-cell myelination by controlling myelin protein gene expression [23] and cholesterol/lipid biosynthesis via the sterol regulatory element binding protein (SREBP) pathway. EGR2/Krox20 mutations in humans are associated with demyelinating or dysmyelinated forms of CMT (CMT1D, CMT4E). Mutations in the zinc finger domain lead to the dominantly inherited form CMT1D. A particular mutation, located in the R1 domain of EGR2/Krox20, accounts for CMT4E.

## 4. Implications in Therapeutical Development

In line of the preceding chapter, cellular, biochemical and molecular phenotypes are different in CMT subtypes, caused by mutations in different genes. This asks the question of developing a drug that could cure all forms of CMT. Therefore, two lines of approaches could be proposed regarding drug development in a specific CMT subtype.

The first possibility is to develop a strategy able to correct the direct consequences of the biochemical/molecular anomaly causing the disorder. This could be achieved using different techniques: small molecules (drugs) treatment, gene therapy, specific antisense, etc. This has been proposed for the CMT1A [24,25,26], CMTX1 [27,28,29], and CMT1B [30] subtypes.

The second possibility is to target the downstream consequences of mutations in a specific gene. For example, different CMT subtypes have been associated with the inflammation of peripheral nerves. As a consequence, a therapeutical strategy has been proposed to treat inflammation and, as a consequence, to stop myelin degradation [31]. However, this option is probably limited to demyelinating CMT subtypes, as these disorders only present neuroinflammation.

Finally, a promising approach will be to develop rehabilitation programs specifically adapted to CMT patients [32]. This aspect has been neglected in the past and should probably be developed.
